# Two NADPH-dependent 2-ketogluconate reductases involved in 2-ketogluconate assimilation in *Gluconobacter* sp. strain CHM43

**DOI:** 10.1128/aem.02501-24

**Published:** 2025-01-29

**Authors:** Sakura Nakashima, Minenosuke Matsutani, Naoya Kataoka, Osao Adachi, Riku Yamashita, Kazunobu Matsushita, Uraiwan Tippayasak, Gunjana Theeragool, Toshiharu Yakushi

**Affiliations:** 1 Joint Degree Program of Kasetsart University and Yamaguchi University, Graduate School of Science and Technology for Innovation, Yamaguchi University 13150 , Yamaguchi, Japan; 2 Department of Food, Aroma and Cosmetic Chemistry, Faculty of Bioindustry, Tokyo University of Agriculture, Hokkaido, Japan; 3 Division of Agricultural Science, Graduate School of Science and Technology for Innovation, Yamaguchi University 13150 , Yamaguchi, Japan; 4 Department of Biological Chemistry, Faculty of Agriculture, Yamaguchi University 98403 , Yamaguchi, Japan; 5 Research Center for Thermotolerant Microbial Resources, Yamaguchi University 13150 , Yamaguchi, Japan; 6 Department of Microbiology, Faculty of Science, Kasetsart University 539116 , Bangkok, Thailand; Shanghai Jiao Tong University, Shanghai, China

**Keywords:** acetic acid bacteria, *Gluconobacter*, 2-ketogluconate, 2-ketogluconate reductase, NADPH

## Abstract

Incomplete oxidation of glucose by *Gluconobacter* sp. strain CHM43 produces gluconic acid and then 2- or 5-ketogluconic acid. Although 2-keto-D-gluconate (2KG) is a valuable compound, it is sometimes consumed by *Gluconobacter* itself via an unknown metabolic pathway. We anticipated that 2KG reductase (2KGR) would be a key enzyme in 2KG metabolism. *GLF_0478* and *GLF_1777* were identified in the genome of strain CHM43, which encode proteins with 70% and 48% amino acid sequence identity, respectively, to the 2KGR of *Gluconobacter oxydans* strain 621H. Constructed mutant derivatives of strain CHM43 lacking *GLF_0478*, *GLF_1777*, or both were examined for their 2KG consumption ability. Strains ∆*GLF_0478* and ∆*GLF_1777* consumed 2KG like the parental strain. However, the double-deletion (∆∆) strain did not consume 2KG at all, although it produced 2KG like the parental strain. Strains ∆*GLF_0478* and ∆*GLF_1777* each showed decreased 2KGR activity compared with the parental strain, and strain ΔΔ lost 2KGR activity. These results suggest that reduction of 2KG catalyzed by GLF_0478 and GLF_1777 is the committed step in 2KG metabolism in *Gluconobacter* sp. strain CHM43. The two 2KGRs showed high activity at neutral pH and lower *K*_M_ values for NADPH than NADH. Results of induction experiments suggest that GLF_0478 is constitutively expressed at a low level but induced by 2KG, and GLF_1777 is also inducible by 2KG but repressed in the absence of an inducer. Our study that characterizes the key genes for 2KG consumption in *Gluconobacter* gives insights for improvement of biological 2KG production systems.

**IMPORTANCE:**

2-Keto-D-gluconate (2KG), a product of incomplete oxidation of glucose by acetic acid bacteria including *Gluconobacter* spp., is used for various purposes, including in the food industry. *Gluconobacter* also consumes 2KG via an unclear metabolic pathway. It is reported that *Pseudomonas* spp. and *Cupriavidus necator* phosphorylate 2KG in the first step of 2KG metabolism, but some enteric bacteria including *Escherichia coli* reduce 2KG. This study evaluated the 2KG consumption ability of a mutant derivative of a strain of *Gluconobacter* that lacks two putative 2KGR-encoding genes. The mutant strain did not consume 2KG at all; the two 2KGRs were each found to catalyze 2KG reduction. It is concluded that reduction of 2KG is the committed step in 2KG metabolism in *Gluconobacter*. The results presented here give insights that might facilitate improvement of 2KG production systems that use *Gluconobacter*.

## INTRODUCTION

2-Keto-D-gluconic acid is a precursor for the synthesis of isoascorbic acid, a valuable antioxidant used in the food processing industry (1), and can be used in the synthesis of a wide variety of chemicals such as hydrophilic triazines, spiro-connected heterocyclics, and pyranoic amino acids (2). 2-Keto-D-gluconate (2KG) can be produced from glucose by enzymatic conversion, microbial fermentation, and resting cell transformation. Microorganisms used in 2KG production include *Gluconobacter* spp. (3, 4), *Acetobacter orleanensis* (formerly *Acetobacter pasteurianus*) (5), *Pseudomonas* sp. (6), *Erwinia* sp. (7), and *Klebsiella* sp. (8). These microorganisms oxidize glucose using a pyrroloquinoline quinone (PQQ)-dependent glucose dehydrogenase (GDH) on the periplasmic side of the cytoplasmic membrane to produce gluconic acid. Gluconic acid is then oxidized to 2KG on the periplasmic side of the membrane by flavin adenine dinucleotide-dependent gluconate 2-dehydrogenase (GADH) (Fig. S1 to S3). These dehydrogenases are linked to the respiratory chain that reduces molecular oxygen to water, which produces proton motive force across the cytoplasmic membrane and in turn drives ATP synthesis (9). Because the metabolic process occurs in the periplasmic space, it does not require transport of substrates and products across the cytoplasmic membrane, and the reaction products accumulate rapidly in the culture medium.

*Gluconobacter* spp. belong to a group of acetic acid bacteria that is characterized by incomplete oxidation of a broad range of sugars and sugar alcohols in the periplasmic space. In addition to 2KG production, *Gluconobacter* spp. oxidize gluconic acid via a PQQ-dependent glycerol dehydrogenase (GLDH; encoded by the genes *sldBA*) on the periplasmic side of the cytoplasmic membrane to produce 5-keto-D-gluconate (5KG) (Fig. S3) (10). *Gluconobacter* sp. strain CHM43, a thermotolerant strain isolated in Thailand (11), produces L-erythrulose from *meso*-erythritol as well as 2KG from glucose (12). Strain CHM43 also produces a low amount of 5KG. Because neither 2KG nor 5KG is metabolized on the cell surface of strain CHM43, they are thought to be transported to, and metabolized in, the cytoplasm. According to genome data, strain CHM43 does not have genes for a phosphotransferase system (13), suggesting that the transport of 2KG and 5KG may not involve phosphorylation. Although 2KG reductases (2KGRs) and 5KG reductases (5KGRs) have been characterized in a wide variety of acetic acid bacteria (14), the metabolic pathway of 2KG in *Gluconobacter* spp. remains uncertain.

*Gluconobacter japonicus* NBRC 3271 effectively consumes 5KG, but a mutant of this strain from which the *gno* gene, encoding NADPH-dependent 5KGR, has been eliminated does not consume 5KG (15). It is reasonably considered that in wild-type *G. japonicus* NBRC 3271, 5KG in the extracellular milieu is transported into the cytoplasm without phosphorylation and reduced to gluconate in an NADPH-dependent manner; the gluconate then enters the pentose phosphate pathway or the Entner–Doudoroff pathway. Because the ability to reduce 2KG has been observed in cell extracts of acetic acid bacteria, there may be a similar metabolic pathway for 2KG. 2KGRs have been purified and characterized from acetic acid bacteria, including *Gluconacetobacter liquefaciens* (formerly *Gluconobacter liquefaciens*) (16, 17), *Acetobacter rancens* (18), *Acetobacter ascendens* (19), and *Gluconobacter oxydans* (20, 21). However, the 2KGRs from acetic acid bacteria have differing catalytic properties. The most notable difference in enzyme activity is gluconate oxidation activity: 2KGRs from *Gluconobacter* spp. are faster than those from *Acetobacter* spp. (14). The physiological roles of 2KGR in 2KG metabolism in *Gluconobacter* spp. have not yet been examined.

In *Erwinia* sp. (22), *Klebsiella* sp. (23), and *Escherichia coli* (24), the key enzymes in the metabolic pathway of 2KG are 2KGR and gluconate kinase. These species assimilate 2KG, and the activity of the two enzymes can be detected in cell extract. Thus, it is considered that in these species, 2KG is transported into the cytoplasm without phosphorylation, reduced to gluconate by oxidizing NADPH, and then phosphorylated to 6-phosphogluconate, which enters the pentose phosphate pathway or the Entner–Doudoroff pathway. Indeed, a mutant strain of *E. coli* lacking the *yiaE* gene encoding 2KGR cannot grow on 2KG as a sole source of carbon and energy, indicating that 2KGR has a crucial role in 2KG assimilation in *E. coli* (24).

In contrast, *Cupriavidus necator* (formerly *Ralstonia eutropha*) and several *Pseudomonas* spp. are proposed to consume 2KG in a process that first involves phosphorylation (Fig. S2) (25–27). The phosphorylation of 2KG, catalyzed by 2KG kinase (KguK), which produces 2-keto-6-phosphogluconate (2K6PG), is considered the committed step in the assimilation of 2KG by these bacteria (28, 29). 2K6PG is then reduced to 6-phosphogluconate by NADPH-dependent 2K6PG reductase (KguD). However, the KguK activity in glucose-grown *Pseudomonas putida* KT2440 cells is moderate (27) and intracellular 2K6PG is undetectable in *P. putida* S12 cells (30). Moreover, the *kguK*-knockout derivative of *Pseudomonas plecoglossicida* strain JUIM01 showed delayed growth on 2KG and 2KG consumption compared with the parental strain but later grew on 2KG and consumed 2KG completely (29). The results suggest the presence of another phosphorylation enzyme than KguK or another metabolic pathway bypassing phosphorylation of 2KG, in *P. plecoglossicida*.

The mechanism by which 2KG is metabolized in *Gluconobacter* is unclear. Thus, in the present study, we attempted to assess the physiological role of 2KGR in 2KG consumption and assimilation in *Gluconobacter* sp. strain CHM43. There are two candidate genes to encode 2KGR in the genome of strain CHM43; we constructed and analyzed gene deletion derivatives and overproduction strains for these candidate genes.

## RESULTS

### Candidates for 2KGR in the strain CHM43 genome

**Fig 1:**
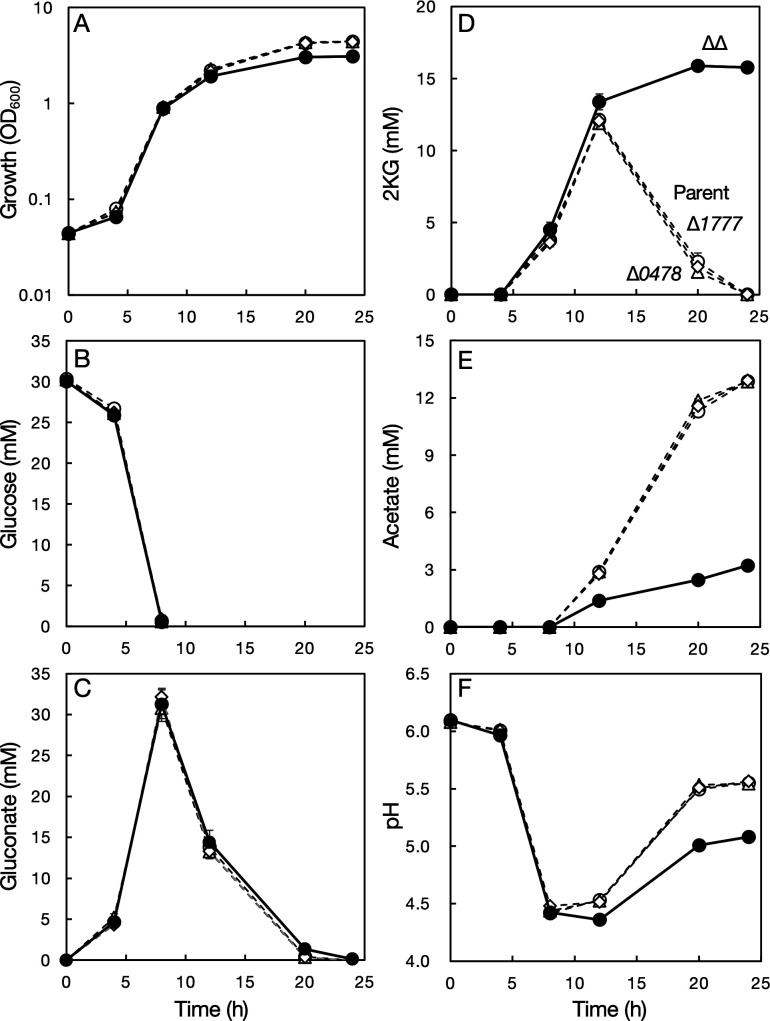
Growth and metabolite profiles of *Gluconobacter* sp. CHM43 derivatives grown on glucose. Four derivatives of *Gluconobacter* sp. CHM43 were cultivated in YPD medium (including 0.5% D-glucose and 0.1 M potassium dihydrogen phosphate, pH 6.0) at 30°C for 24 h with shaking (200 rpm). Open circles, strain TORI3 (Δ*sldBA*, “Parent”); open triangles, strain SKR204 (Δ*sldBA* ∆*GLF_0478*, “∆*0478*”); open diamonds, strain SKR217 (Δ*sldBA* ∆*GLF_1777*, “∆*1777*”); closed circles, strain SKR247 (Δ*sldBA* ∆*GLF_0478* ∆*GLF_1777*, “∆∆”). (A) Cell growth; (B) glucose; (C) gluconate; (D) 2-ketogluconate (2KG); (E) acetate; (F) pH. Mean values and standard deviations (error bars) are shown from triplicate cultures.

This study focused on 2KG metabolism in *Gluconobacter* sp. CHM43. We began with the mutant derivative TORI3 that lacks the *sldBA* genes that encode the GLDH responsible for 5KG production. This therefore eliminated production of 5KG and the need for complex metabolite analysis (31). When strain TORI3 (∆*sldBA*) was grown on glucose, glucose was converted to gluconic acid and to 2KG. The 2KG was completely consumed by the TORI3 cells within 24 h if the pH of the culture medium was maintained above 4 (Fig. 1). If the pH of the medium was not controlled, the pH decreased to below 4 and the TORI3 cells did not consume 2KG. Other *Gluconobacter* spp., such as *G. oxydans* 621H (32), *G. oxydans* DSM2003 (33), and *G. japonicus* CGMCC 1.49 (34), were reported to hardly consume 2KG. Therefore, we anticipated that strain TORI3 is suitable for examination of 2KG consumption by *Gluconobacter* cells.

The gene encoding 2KGR of *G. oxydans* 621H (*GOX0417*) was reported by Saichana et al. (20) and Rauch et al. (21). We searched for homologous gene(s) in the genome of *Gluconobacter* sp. strain CHM43 (35). GLF_0478 and GLF_1777 have 70% and 48% amino acid sequence identity to GOX0417, respectively. Homologs of GLF_0478 and GLF_1777 were respectively retrieved from public genome data for Acetobacteraceae (Table S1), and the phylogeny of the two candidate 2KGRs was examined (Fig. S4). In our phylogenetic tree, the 2KGR homologs from Acetobacteraceae formed three clades; both 2KGR candidates from *Gluconobacter* sp. strain CHM43 were in the same clade. We have reported purification of 2KGR from *Ga. liquefaciens* NBRC 12388 (16) (formerly *G. liquefaciens* IFO 12388) and *A. pasteurianus* NBRC 3299 (19) (formerly *A. ascendens* IFO 3299). One 2KGR homolog was exclusively found in each of the genomes of *Ga. liquefaciens* DSM 5603 (QQAW00000000; synonym of NBRC 12388) and *A. pasteurianus* NBRC 3299 (BDEZ00000000): GLI01_12690 and NBRC3299_0848, respectively. It is likely that these genes encode the 2KGRs. They are in a different clade in the phylogenetic tree (Fig. S4) from the putative 2KGRs identified in *Gluconobacter* sp. CHM43.

### 2KG consumption and assimilation by derivatives of strain CHM43

Starting with strain TORI3 (∆*sldBA*), derived from *Gluconobacter* sp. strain CHM43, we constructed gene deletion mutants for the two putative 2KGR-encoding genes, *GLF_0478* and *GLF_1777*: strains SKR204 (∆*sldBA* ∆*GLF_0478*), SKR217 (∆*sldBA* ∆*GLF_1777*), and SKR247 (∆*sldBA* ∆*GLF_0478* ∆*GLF_1777*), which are hereafter referred to as ∆*GLF_0478*, ∆*GLF_1777*, and ∆∆, respectively. The two single-mutant strains, ∆*GLF_0478* and ∆*GLF_1777*, showed similar growth and metabolite profiles to those of the parental strain TORI3 (∆*sldBA*) (Fig. 1). However, the double-mutant strain ∆∆ grew less well than the parental strain in the late phase of growth (Fig. 1A). The double-mutant strain ∆∆ did not consume 2KG at all (Fig. 1D), although the glucose and gluconate profiles of all the bacterial strains were similar to each other (Fig. 1B and C). These results indicate that the two candidate 2KGRs in *Gluconobacter* sp. CHM43 are independently involved in 2KG consumption and either one of them is sufficient to consume 2KG. Acetate was increased in the late phase of growth concomitantly with 2KG consumption by the parental, ∆*GLF_0478*, and ∆*GLF_1777* strains. The ∆∆ strain produced less acetate, presumably because 2KG consumption did not occur (Fig. 1E). For the parental, ∆*GLF_0478*, and ∆*GLF_1777* strains, the pH was decreased in the early phase of growth due to gluconate production and increased later because of assimilation of gluconate or 2KG. The level of pH increase was modest in the ∆∆ strain presumably because of the lack of 2KG consumption (Fig. 1F).

**Fig 2:**
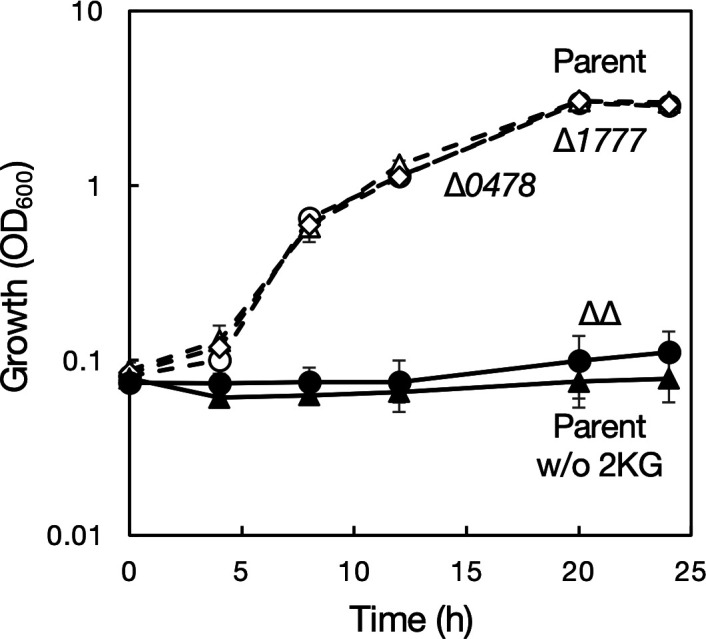
Growth of *Gluconobacter* sp. CHM43 derivatives on 2-ketogluconate. Four derivatives of *Gluconobacter* sp. CHM43 were cultivated in 2KG medium (including 26 mM 2-ketogluconate and 0.1 M potassium dihydrogen phosphate) at 30°C for 24 h with shaking (200 rpm). Open circles, strain TORI3 (Δ*sldBA*, “Parent”); open triangles, strain SKR204 (Δ*sldBA* ∆*GLF_0478*, “∆*0478*”); open diamonds, strain SKR217 (Δ*sldBA* ∆*GLF_1777*, “*∆1777*”); closed circles, strain SKR247 (Δ*sldBA* ∆*GLF_0478* ∆*GLF_1777*, “∆∆”); closed triangles, strain TORI3 grown on medium without 2KG. Mean values and standard deviations (error bars) are shown from triplicate cultures.

The 2KG was prepared as described in the Materials and Methods section for cell growth experiments and enzyme assays. The single-mutant strains ∆*GLF_0478* and ∆*GLF_1777* grew as well as the parental strain TORI3 on 2KG. However, the ∆∆ strain failed to grow on 2KG, and its growth behavior was similar to that of the parental strain in medium without 2KG (Fig. 2). These results indicate that the two candidate 2KGRs in *Gluconobacter* sp. CHM43 have a pivotal role in 2KG assimilation.

### Induction of 2KGRs

**Fig 3:**
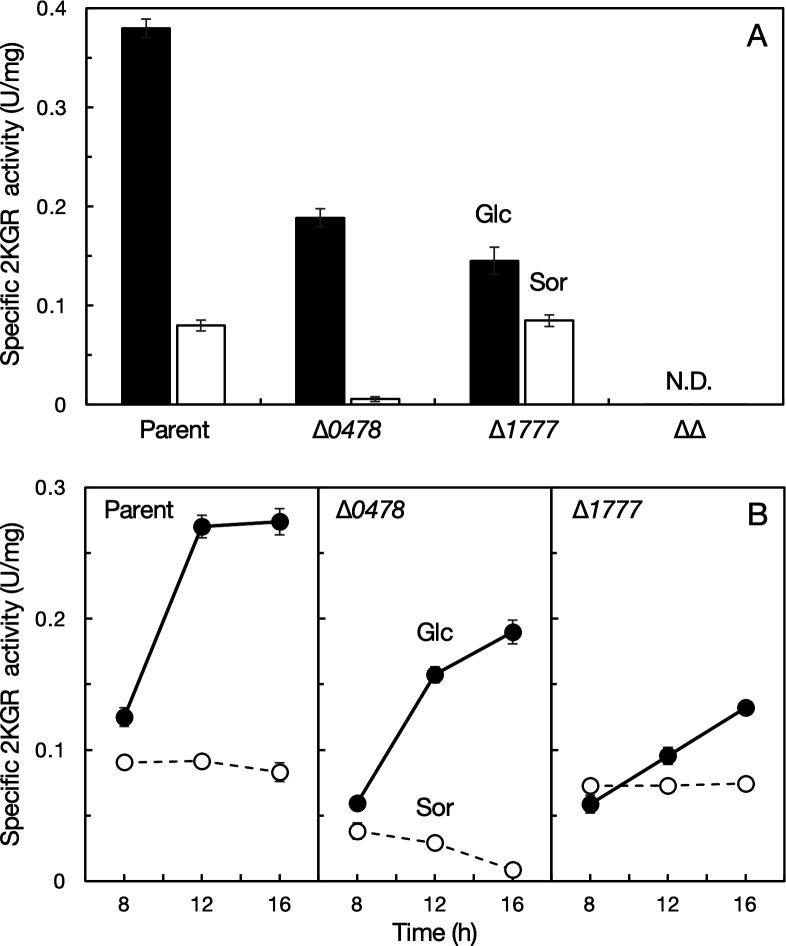
Induction of 2-ketogluconate reductases. (**A**) Four derivatives of *Gluconobacter* sp. CHM43, strains TORI3 (Δ*sldBA*, “Parent”), SKR204 (Δ*sldBA* ∆*GLF_0478*, “∆0478”), SKR217 (Δ*sldBA* ∆*GLF_1777*, “∆1777”), and SKR247 (Δ*sldBA* ∆*GLF_0478* ∆*GLF_1777*, “∆∆”), were cultivated in YPD medium (containing glucose, “Glc” black bars) or YPS medium (containing sorbitol, “Sor” white bars) at 30°C for 16 h with shaking. (**B**) Three derivatives of *Gluconobacter* sp. CHM43, strains TORI3 (Parent, left), SKR204 (∆*0478*, center), and SKR217 (∆*1777*, right), were cultivated in YPD medium (Glc, black circles) or YPS medium (Sor, white circles) at 30°C for 8, 12, and 16 h with shaking. 2KGR activity in the cell-free extract was measured using NADPH. Mean values and standard deviations (error bars) are shown from triplicate enzyme assays. N.D., not detected.

The NADPH-dependent 2KGR activities of the parental strain TORI3 and strains ∆*GLF_0478*, ∆*GLF_1777*, and ∆∆ grown on glucose were examined (Fig. 3A, black bars). The double-mutant ∆∆ strain did not show detectable 2KGR activity. These results indicate that GLF_0478 and GLF_1777 contribute most of the 2KGR activity of *Gluconobacter* sp. CHM43 and the presence of an additional 2KGR is unlikely. GLF_0478 and GLF_1777 showed comparable 2KGR activity *in vitro*, which may account for functional redundancy of the two 2KGRs in 2KG consumption *in vivo* (Fig. 1).

The highest activity was detected in the cell-free extract of the parental strain. Approximately 49% and 38% of the 2KGR activity of the parental strain were detected in strains ∆*GLF_0478* and ∆*GLF_1777*, respectively, grown for 16 h (Fig. 3A, black bars). Activities of 2KGR were higher in the parental, ∆*GLF_0478*, and ∆*GLF_1777* cells grown in glucose-containing medium than in sorbitol-containing medium (Fig. 3A, white bars). In particular, 2KGR activity in strain ∆*GLF_0478* was strongly induced in the glucose medium (approximately 34-fold), suggesting that expression of GLF_1777 is tightly repressed by unknown mechanism. In contrast, induction of 2KGR activity in strain ∆*GLF_1777* grown on glucose was moderate. These data suggest that repression of GLF_0478 is not strict in the absence of glucose and its derivatives and its expression is inducible but not strong in glucose medium. In the early stage of the growth on glucose (Fig. 3B, black), the 2KGR activities of the three strains grown for 8 h were much lower than those grown for 16 h. The activities of three strains were increased as the cultures on glucose proceed. On the other hand, the activity of strain ∆*GLF_0478* was decreased as the culture on sorbitol proceeds (Fig. 3B, white), suggesting a tight repression of *GLF_1777*. Because the early stage of the growth at 8 h is a 2KG production phase but the late stage at 16 h is a 2KG consumption phase (Fig. 1), these results suggest that 2KG induces expression of the two 2KGRs.

Glucose is converted to gluconate and 2KG, and therefore, we attempted to understand which compound is responsible for induction of the 2KGRs. In strain UCD1 (a *gdhM*-deficient derivative of strain CHM43), glucose is not converted into gluconate by the cell surface oxidation system. In strain TERA3 (∆*sldBA* ∆*gndFGH*), gluconate is not converted into ketogluconates in the periplasmic space. We examined 2KGR activity in strain TORI3 (∆*sldBA*) grown on 2KG, strain UCD1 (∆*gdhM*) grown on glucose, and strain TERA3 grown on gluconate (Fig. S5). The results indicate that 2KG strongly induces 2KGR expression. The gene organization of the adjacent regions to the genes *GLF_0478* and *GLF_1777* (Fig. S6) implies that the expression of *GLF_1777* is regulated by the putative transcriptional regulator(s) GLF_1772 and/or GLF_1773. The expression of *GLF_0478* would be regulated by some global transcriptional regulator or another type of regulatory system.

### Properties of the 2KGRs

For enzymatic characterization, we attempted to construct overexpression strains for the two 2KGRs. For overexpression of GLF_0478, we constructed strain SKR117 (∆*GLF_1777*) harboring pSKR58 (*GLF_0478*^+^) and GLF_1777, strain SKR104 (∆*GLF_0478*) harboring pSKR57 (*GLF_1777*^+^). GLF_1777 was prone to aggregate in the conditions in our initial experimental setup; therefore, 300 mM KCl was added into the buffers for enzyme preparation to avoid aggregation of GLF_1777.

**Fig 4:**
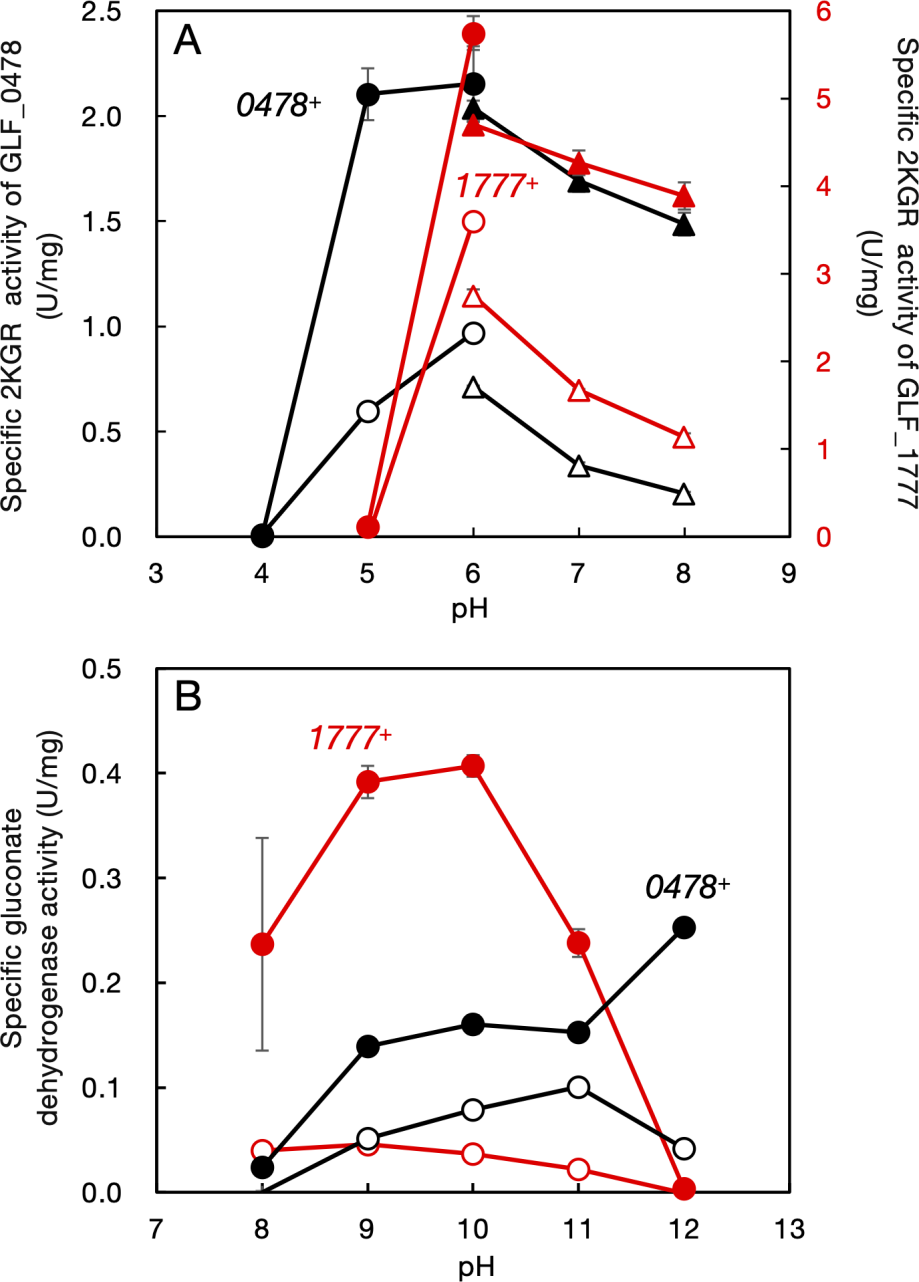
Effect of pH on 2-ketogluconate reductase and gluconate dehydrogenase activities. Two derivatives of *Gluconobacter* sp. CHM43, strain SKR117 (Δ*GLF_1777*) harboring pSKR58 (*GLF_0478*^+^), shown in black (“*0478*^+^”), and strain SKR104 (∆*GLF_0478*) harboring pSKR57 (*GLF_1777*^+^), shown in red (“*1777*^+^”), were cultivated in YPD medium at 30°C for 16 h with shaking (200 rpm). Soluble cell-free extract was prepared and used for 2KGR assay (**A**) with NADPH (closed symbols) or NADH (open symbols) as the electron donor and also used for gluconate dehydrogenase assay (**B**) with NADP^+^ (closed symbols) or NAD^+^ (open symbols) as the electron acceptor. (A) Na^+^-acetate (circles) and K^+^-phosphate (triangles) were used as the buffer for enzyme assays. (B) Na^+^-glycine was used as the buffer for enzyme assays. Mean values and standard deviations (error bars) are shown from triplicate enzyme assays.

The activity of 2KGR was detected in the soluble fractions of cell extracts. NADPH (rather than NADH) was the preferable cofactor for the two 2KGRs: the reaction rates with NADH for the two 2KGRs were approximately half of those with NADPH (Fig. 4). The two 2KGRs showed similar pH dependencies between 6 and 8; their activities at pH 5 were different. At pH 5, GLF_0478 showed similar activity to that at pH 6, but GLF_1777 showed almost no activity at pH 5 (Fig. 4). The *K*_M_ values for 2KG of the two 2KGRs were similar to those reported previously (16, 18, 19, 21). GLF_1777 showed a higher *K*_M_ value for 2KG than GLF_0478, but the *K*_M_ value was dependent on the cofactor (Table 1). The *K*_M_ value for NADPH was much lower than that for NADH for the two 2KGRs (Table 1; Fig. S7), suggesting that they have higher affinity for NADPH than NADH.

**TABLE 1:** Characteristics of GLF_0478, GLF_1777, and previously reported 2-ketogluconate reductases^*a*^

	*Acetobacter pasteurianus* NBRC 3299	*Acetobacter* sp. NBRC 3298	*G. liquefaciens* IFO 12388	GOX0417	GLF_0478	GLF_1777
Molecular mass (protomer)	120 kDa (40 kDa)	120 kDa (15 kDa)	110 kDa	(33 kDa)	(33 kDa)	(33 kDa)
2KG reduction						
Optimum pH	6.0	7.0	6.5	–	6	6
*K*_M_ for 2KG (mM) with NADPH	5.3	0.86	6.6	5.2	8.5 ± 1	16 ± 2
With NADH	*^–a^*	–	–	–	12 ± 0.9	7.5 ± 0.8
*K*_M_ for NADPH (µM)	7.4	10	15.2	<10	13 ± 3	3.0 ± 0.4
*K*_M_ for NADH (µM)	–	–	–	51.5	150 ± 10	100 ± 9
GA oxidation						
Optimum pH	10.5	12.0	10.5	–	12	9–10
*K*_M_ for GA (mM)	4.4	2.4	13	–	–	–
References	(19)	(18)	(16)	(20, 21)	This study	This study

^*a*^ –, no data.

The reverse reaction of 2KGR, gluconate oxidation, was examined using the two 2KGRs. The highest gluconate oxidation activity was detected at pH 10 for GLF_1777 and at pH 12 for GLF_0478 (Fig. 4). High gluconate oxidation activity at alkaline pH was also observed for 2KGRs of *Acetobacter* sp. (18) and *A. pasteurianus* (19). The rates of gluconate oxidation by GLF_1777 and GLF_0478 were approximately one-tenth those for 2KG reduction (Fig. 4).

In *Pseudomonas* spp., NADPH-dependent reduction of 2K6PG is involved in 2KG consumption (26, 27, 29, 30). We examined a possibility of 2K6PG reduction by GLF_0478 and GLF_1777. However, 2K6PG is not commercially available in this moment. Therefore, 6-phosphogluconate oxidation was investigated using the two 2KGRs, because the two enzymes showed significant gluconate oxidation activity, which is unlikely to be physiologically relevant. The host *Gluconobacter* cells showed the activity of 6-phosphogluconate dehydrogenase (presumably decarboxylating enzyme [EC 1.1.1.44]) at neutral pH, but it was almost undetectable at pH 10 and 12. However, oxidation of 6-phosphogluconate (the reverse reaction of 2K6PG reduction) was not elevated upon expression of the two 2KGRs, suggesting that they do not catalyze reduction of 2K6PG.

## DISCUSSION

*Gluconobacter* spp. produce 2KG from glucose in a high yield and 5KG in a low yield, simultaneously. Previous studies identified the enzymes responsible for 2KG and 5KG production. Then, genetic engineering strategies enabled the creation of mutant *Gluconobacter* derivatives lacking the 5KG- and 2KG-producing enzymes (4, 36). To enhance the yield of 2KG and 5KG, such as for industrial production purposes, it is beneficial to eliminate the genes encoding enzymes that degrade them. Recently, we created a mutant *G. japonicus* strain lacking the *gno* gene for NADPH-dependent 5KGR; the ∆*gno* strain produced 5KG from glucose with much higher yield than the parental strain (15). However, degradation of 2KG in *Gluconobacter* spp. is not well understood. Indeed, *Gluconobacter* cells rarely consume 2KG in conditions where 2KG is produced. Here, we found conditions in which *Gluconobacter* sp. CHM43 strain consumes 2KG well when grown on glucose; therefore, we attempted to understand the mechanism of 2KG consumption in strain CHM43.

We previously reported that several strains of acetic acid bacteria possess NADPH-dependent 2KGR (16, 18, 19). The best-characterized 2KGR of acetic acid bacteria at the molecular level is GOX0417 from *G. oxydans* 621H. Mining the genome of strain CHM43, two candidate 2KGR-encoding genes were found, *GLF_0478* and *GLF_1777*, with amino acid sequence identities of 70% and 48% with GOX0417, respectively. The ∆*GLF_0478* and ∆*GLF_1777* single gene-deletion strains did not show any defects in 2KG consumption compared with the parental strain (∆*sldBA*) that does not produce 5KG. However, the double-mutant strain did not consume 2KG at all. These data indicate that genes *GLF_0478* and *GLF_1777* are involved in 2KG consumption, that is, the two 2KGRs are functionally redundant and either one of them is enough for 2KG consumption, and reduction of 2KG is the committed step in 2KG degradation in strain CHM43 (Fig. S3). Although *G. oxydans* 621H possesses the *GOX0417* gene in the genome as the sole gene for 2KGR homolog and *G. japonicus* NBRC 3271 does the *AA3271_0639* gene for 2KGR, these microorganisms do not consume 2KG (32, 37). The 2KG-utilizing systems in *G. oxydans* 621H and *G. japonicus* NBRC 3271 may not work to consume 2KG, even though the 2KGRs are functional. The *GOX0417* and *AA3271_0639* genes are related to *GLF_0478*. Therefore, we speculate that strain CHM43 maintains the “second” 2KG utilization system including GLF_1777 for 2KG consumption. Transporter genes are found in the proximity of *GLF_0478* and *GLF_1777*, in the CHM43 genome, but genes for transcriptional regulator and outer membrane porin are found only in the proximity of *GLF_1777*. Investigating the role of the genes near *GLF_0478* and *GLF_1777* may provide insights into the reason why strain CHM43 possesses the two 2KGRs.

Expression of the two 2KGRs in strain CHM43 was enhanced when the cells were grown in the presence of 2KG (Fig. 3; Fig. S5). 2KGR is involved in assimilation of 2KG (Fig. 2), which is the incomplete oxidation product by strain CHM43 at the cell surface using the membrane-bound dehydrogenases from glucose via gluconate as the intermediate (Fig. S3). In the early stage of the growth with low 2KG levels, the expression levels of 2KGR were low. However, in the late stage of the growth with accumulated 2KG, the expression levels of 2KGR were elevated (Fig. 3). Thus, it is plausibly speculated that strain CHM43 oxidizes glucose to 2KG using the cell-surface oxidation system to obtain energy in the early phase of growth, and then, it consumes 2KG to obtain carbon source by inducing the 2KG assimilation system including 2KGR in the late phase of growth.

In addition to levels of the 2KGR-encoding gene repression in sorbitol medium, those of the 2KGR-encoding gene expression in glucose medium were different for the two 2KGR genes. The organization of the genes surrounding each 2KGR-encoding gene in the genome is also different (Fig. S6). Two genes for LacI family transcriptional regulator GLF_1772 and GLF_1773 are found in the proximity of *GLF_1777*, but no genes for the putative transcriptional regulator are found near *GLF_0478*. The *Pseudomonas putida* PtxS transcriptional regulator, a LacI family, binds to the promoter region of the *kgu* operon for 2KG utilization to repress the transcription (38). The PtxS protein is released from the target DNA upon binding with 2KG in *P. putida*. It is reasonable to speculate that the regulatory mechanisms of expression of the *GLF_0478* and *GLF_1777* genes have evolved independently and they warrant further investigation.

By constructing mutant *Gluconobacter* strains that highly but solely expressed either 2KGR (i.e., GLF_0478 or GLF_1777), the properties of the two 2KGRs were characterized. The two enzymes showed higher catalytic rates with NADPH than with NADH and lower *K*_M_ values for NADPH than for NADH. Thus, both of them prefer NADPH as the cofactor. The two enzymes catalyze not only reduction of 2KG but also oxidation of gluconate in the presence of NADP^+^ or NAD^+^. We attempted to detect oxidation of 6-phosphogluconate by the two 2KGRs, the reverse reaction of 2K6PG reduction, but we failed. Thus, it might be concluded that the two 2KGRs do not catalyze 2K6PG reduction. However, determination of this issue would require precise investigations using the substrate 2K6PG. GLF_0478 and GLF_1777 reduced modestly 5-keto-D-gluconate (5KG), another major product by incomplete oxidation of glucose by *Gluconobacter* spp., in our experimental setup: approx. 2%–3% of the 2KG reduction rate, which is consistent with the previous reports (18, 19). 2KGRs of *Acetobacter*, *Gluconacetobacter*, and *Gluconobacter* show similar substrate spectrum (19), even though they are phylogenetically different from each other (Fig. S4). We speculate that GLF_0478 and GLF_1777 have a similar substrate spectrum.

Acetic acid bacteria, including *Gluconobacter* sp., are regarded as GRAS (generally recognized as safe). Therefore, 2KG production by *Gluconobacter* spp. has several advantages over that by other microorganisms, such as for applications in foods. Above pH 4, *Gluconobacter* sp. strain CHM43 consumed 2KG, while below pH 4, it did not, indicating that the cultivation conditions are crucial for a high-level 2KG production process. Therefore, we consider that characterization of the 2KG consumption pathway of *Gluconobacter* has implications for possible application in 2KG production processes. Blocking the metabolic pathway(s) for 2KG consumption may help maintain high-level 2KG production.

## MATERIALS AND METHODS

### Chemicals

NAD(P)H, NAD(P)^+^, and yeast extract were supplied by Oriental Yeast (Tokyo, Japan). Hipolypepton was from Shiotani M.S. (Amagasaki, Japan). 2KG was prepared by using resting *Gluconobacter* cells as described in the section “Preparation of 2KG.” All other chemicals used in this study were commercial products.

### Bacterial strains, plasmids, and culture conditions

The bacterial strains and plasmids used in this study are listed in Table 2. *Gluconobacter* strains were grown on YPS medium (3 g yeast extract, 3 g Hipolypepton, and 50 g D-sorbitol per liter), YPD medium (3 g yeast extract, 3 g Hipolypepton, 5 g glucose, and 13.6 g potassium dihydrogen phosphate per liter, pH 6.0 [adjusted with KOH]), G-GA medium (3 g yeast extract, 3 g Hipolypepton, 10 g glucose, and 10 g Na^+^-gluconate per liter), GA medium (3 g yeast extract, 3 g Hipolypepton, and 5 g Na^+^-gluconate per liter, pH 4.5 [adjusted with phosphoric acid]), or 2 KG medium (3 g yeast extract, 3 g Hipolypepton, 26 mmol 2 KG, and 100 mmol potassium dihydrogen phosphate per liter). *E. coli* strain DH5α was used for plasmid construction (39). *E. coli* strain HB101 harboring pRK2013 was used for triparental mating (40). *E. coli* strains were grown on modified Luria–Bertani medium (10 g Hipolypepton, 5 g yeast extract, and 5 g NaCl per liter, pH 7.0 [adjusted with NaOH]). Ampicillin, tetracycline, and kanamycin were used at final concentrations of 50 µg mL^−1^, 10 µg mL^−1^, and 50 µg mL^−1^, respectively, for *E. coli*, and 500 µg mL^−1^, 10 µg mL^−1^, and 50 µg mL^−1^, respectively, for *Gluconobacter*.

**TABLE 2:** *Gluconobacter* strains and plasmids used in this study

Strain or plasmid	Relevant characteristics	Source or reference
*Gluconobacter* sp. strains		
CHM43	Wild type	(11)
TORI3	CHM43 ∆*sldBA* (∆GLDH)	(31)
SKR104	CHM43 ∆*GLF_0478*	This study
SKR117	CHM43 ∆*GLF_1777*	This study
SKR204	TORI3 ∆*GLF_0478* (∆GLDH ∆GLF_0478)	This study
SKR217	TORI3 ∆*GLF_1777* (∆GLDH ∆GLF_1777)	This study
SKR247	SKR204 ∆*GLF_1777* (∆GLDH ∆GLF_0478 ∆GLF_1777)	This study
TERA3	TORI3 ∆*gndFGH* (∆GLDH ∆GADH)	This study
UCD1	CHM43 ∆*gdhM* (∆GDH)	This study
Plasmids		
pKOS6b	Suicide vector, *mob codAB*, Km^R^	(41)
pKOS6bTc	Suicide vector, *mob codAB*, Tc^R^	This study
pBBR1MCS-4	Broad host range vector, *mob*, Ap^R^	(42)
pCM62	Broad host range vector, *mob*, Tc^R^	(43)
pSHO8	pBBR1MCS-4, a 0.7 kb DNA fragment of promoter region of the *adhAB* gene	(44)
pSKR28	pKOS6bTc, a 1.8 kb DNA fragment of a ∆*GLF_0478* allele	This study
pSKR27	pKOS6bTc, a 1.9 kb DNA fragment of a ∆*GLF_1777* allele	This study
pJ336	pKOS6b, a 1.4 kb DNA fragment of a ∆*gndFGH* allele	This study
pJ793	pKOS6b, a 1.4 kb DNA fragment of a ∆*gdhM* allele	This study
pSKR58	pSHO8, a 1.1 kb DNA fragment of the *GLF_0478* gene	This study
pSKR57	pSHO8, a 1.0 kb DNA fragment of the *GLF_1777* gene	This study

### Determination of glucose, gluconate, 2KG, and acetate

Culture was taken and centrifuged at 12,000 × *g* for 5 min at 4°C to remove cells. The supernatant was filtered through a 0.45 µm filter (Millipore, Billerica, MA, USA) and analyzed by high-performance liquid chromatography (HPLC). Samples were run on a Pb^2+^-coordinated cation exchange column (SUGAR SP810, 8.0 mm i.d. × 300 mm; Shodex, Showa Denko KK, Kawasaki, Japan) at 80°C with deionized water as the mobile phase at a flow rate of 0.5 mL min^−1^ or on an ion exclusion column (RSpak KC-811, 8.0 mm i.d. × 300 mm; Shodex, Showa Denko KK) at 60°C with 0.1% (wt/vol) phosphoric acid as the mobile phase at a flow rate of 0.4 mL min^−1^. Glucose was detected using a refractive index detector, and gluconate, 2KG, and acetate were detected using a diode array detector at 210 nm. Gluconate was also determined enzymatically using an enzymatic determination kit for D-gluconic acid/D-glucono-δ-lactone (R-BioPharm, Darmstadt, Germany).

### Preparation of 2KG

Strain TORI3 (Δ*sldBA*), which cannot produce 5KG, was inoculated into 100 mL of YPS medium in 500 mL Erlenmeyer flasks and cultivated at 30°C overnight with shaking at 200 rpm. Five milliliters of this preculture was transferred to 500 mL of G-GA medium and incubated at 30°C for 48 h with shaking at 200 rpm. The cells were collected by centrifugation (8,000 × *g*, 10 min, 25°C) and washed with 2.5% glucose solution. The cell suspension with 2.5% glucose was added to 300 mL of 5% glucose in a 500 mL jar fermenter to give an OD_600 nm_ of 10. Resting cell reaction was carried out at 30°C with agitation at 500 rpm and an air flow rate of 1.0 vvm, and the pH was kept at 4.0 by applying 5 M KOH. After reaction for 48 h, the cells were removed by centrifugation (8,000 × *g*, 10 min, 4°C) to obtain the supernatant.

Then, the pH of the supernatant was adjusted to 6.0 with KOH and it was filtered with a 0.44 µm pore membrane. The filtrate was applied to a 200 mL anion exchange phosphate-charged Dowex 1 × 4 column equilibrated with ion-exchanged water. 2KG adsorbed onto the ion exchange column and was eluted with a concentration gradient of phosphoric acid (0–0.1 M). The elution was continued with 0.1 M phosphoric acid. Levels of glucose, gluconate, 2KG, and other compounds were estimated by HPLC using an organic acid column. The fractions containing 2KG were pooled, the pH of the pooled solution was adjusted to 5.0 with KOH, and it was frozen at −20°C. 2KG was concentrated by repeated freezing and thawing. Then finally, the 2KG was concentrated by freeze drying. Compounds other than 2KG were not detected in the final preparation by HPLC analysis. The 2KG concentration in the final preparation was determined by HPLC with a standard of authentic 2KG hemicalcium salt (Sigma-Aldrich, Taufkirchen, Germany). Phosphate was determined by the Bartlett assay, in which dipotassium hydrogen phosphate was used as the standard (45).

### Construction of plasmids

The oligonucleotides for use as DNA primers are listed in Table 3. We constructed a derivative plasmid of the suicide vector pKOS6b carrying different antibiotic resistance. The plasmid pKOS6b except for the kanamycin resistance gene was amplified with Herculase II fusion DNA polymerase (Stratagene, CA) and the primer pair pK19daphII-R and pK19daphII-F. The tetracycline resistance gene was amplified from pCM62 with primer pair pCM-tetR-3(−) and pCM-tetA-3(−) (43). The two PCR products were ligated to construct pKOS6bTc.

**TABLE 3:** Oligonucleotide primers used in this study

Oligonucleotide	Sequence (5′–3′)	Objective
pK19daphII-R	gcgaaacgatcctcatcctg	pKOS6bTc
pK19daphII-F	gcgggactctggggttcgct	pKOS6bTc
pCM-tetR-3(−)	aaagggcctcgtgatacgcc	pKOS6bTc
pCM-tetA-3(−)	ttccacgatcagcgatcggc	pKOS6bTc
CHM-D-GLF_0478-Xba(+)	tctagaaaggctctctccagttgc	∆*GLF_0478*
CHM-ex-GLF_0478-HinRI(+)	aagcttgaattctcatctgtcttacctg	*GLF_0478* ^+^
CHM-D-GLF_0478-fsn(+)	ccgacattctggcaatcgatatcaatccagagtttttcac	∆*GLF_0478*
CHM-D-GLF_0478-fsn(−)	gtgaaaaactctggattgatatcgattgccagaatgtcgg	∆*GLF_0478*
CHM-ex-GLF_0478-Xba(−)	tctagaggtcagaggattggggtc	*GLF_0478* ^+^
CHM-D-GLF_0478-RI(−)	gaattcgcttttactgctgctgc	∆*GLF_0478*
CHM-D-GLF_1777(+)	agtctgcttcctccctctag	∆*GLF_1777*
CHM-ex-GLF_1777-Hin(+)	aagcttggcccattagcgcctg	*GLF_1777* ^+^
CHM-D-GLF_1777-fsn(+)	gaaatcgaagctattcttgatatcgccaatctggccgcac	∆*GLF_1777*
CHM-D-GLF_1777-fsn(−)	gtgcggccagattggcgatatcaagaatagcttcgatttc	∆*GLF_1777*
CHM-ex-GLF_1777-Xba(−)	tctagagttattgcactggcg	*GLF_1777* ^+^
CHM-D-GLF_1777(−)	agacgccggtataaagcttg	∆*GLF_1777*
CHM-gndF-5-Hin(+)	aagcttggatgacggatgccatc	∆*gndFGH*
CHM-∆gndF-5-T22(−)	atgcatctgtcgatcaataaattc	∆*gndFGH*
CHM-gndH-3-Xba(−)	tctagactgggtctttttcaggcg	∆*gndFGH*
CHM-∆gdhM-5-Sal(+)	gtcgacgcttcttgttgcgacgg	∆*gdhM*
CHM-∆gdhM-5-Nco(−)	ccatggccgggaggatgtgctc	∆*gdhM*
CHM-∆gdhM-3-Nco(+)	ccatggcaggaccgtctgcc	∆*gdhM*
CHM-∆gdhM-3-Kpn(−)	ggtacctctcctgacatgcgcag	∆*gdhM*

The upstream and downstream regions of the *GLF_0478* gene from *Gluconobacter* sp. CHM43 were amplified from genomic DNA of strain CHM43 using Herculase II fusion DNA polymerase (Stratagene, CA) and primer pairs CHM-∆GLF_0478-Xba(+) and CHM-∆GLF_0478-fsn(−) and CHM-∆GLF_0478-fsn(+) and CHM-∆GLF_0478-RI(−), respectively. The two PCR products of approximately 0.9 kb each were fused by PCR using primers CHM-∆GLF_0478-Xba(+) and CHM-∆GLF_0478-RI(−). The resulting 1.8 kb PCR product was digested with *Xba*I and *Eco*RI and ligated with pKOS6bTc treated with *Xba*I and *Eco*RI to construct pSKR28, a deletion plasmid for the *GLF_0478* gene. The deletion plasmid for the *GLF_1777* gene, pSKR27, was constructed in a similar way using primers CHM-∆GLF_1777(+), CHM-∆GLF_1777-fsn(−), CHM-∆GLF_fsn(+), and CHM-∆GLF_1777-Hin(−). pJ336, a gene deletion plasmid for the *gndFGH* (*GLF_1586–4*) genes encoding GADH was constructed as follows. A 5′-flanking region of the *gndF* gene was amplified by PCR using primer set CHM-gndF-5-Hin(+) and CHM-∆gndF-5-T22(−). The PCR product was digested with *Hin*dIII and *Eco*T22I to obtain the 5′-flanking region as a 0.7 kb fragment. The *gndFGH* genes and their proximal region were amplified by PCR using primers CHM-gndF-5-Hin(+) and CHM-gndH-3-Xba(−). Then, the PCR product was digested with *Eco*T22I and *Xba*I to obtain a 0.7 kb DNA fragment of the 3′-flanking region. These two fragments were inserted into the *Hin*dIII and *Xba*I sites of pKOS6b to construct pJ336, which carries an in-frame deletion allele of the *gndFGH* genes. pJ793, a gene deletion plasmid for *gdhM* (*GLF_0328*), which encodes PQQ-dependent glucose dehydrogenase, was constructed by a similar procedure to that for the *gndFGH-*deletion plasmid but using primers CHM-∆gdhM-5-Sal(+), CHM-∆gdhM-5-Nco(−), CHM-∆gdhM-3-Nco(+), and CHM-∆gdhM-3-Kpn(−) (Table 3).

Genes *GLF_0478* and *GLF_1777* were amplified from genomic DNA of strain CHM43 by using Herculase II fusion DNA polymerase and the primer pairs CHM-ex-GLF_0478-HinRI(+) and CHM-ex-GLF_0478-Xba(−) and CHM-ex-GLF_1777-Hin(+) and CHM-ex-GLF_1777-Xba(−), respectively. The amplified 1.1 kb and 1.0 kb PCR products for *GLF_0478* and *GLF_1777*, respectively, were digested with *Hin*dIII and *Xba*I. The fragments were ligated with pSHO8 treated with *Hin*dIII and *Xba*I to construct pSKR58 (*GLF_0478*^+^) and pSKR57 (*GLF_1777*^+^), respectively.

### Construction of bacterial strains

Gene deletion derivatives of *Gluconobacter* sp. strain CHM43 were constructed using suicide plasmids as described previously (31). *Gluconobacter* sp. strain CHM43 was transformed by electroporation using a GenePulser (Bio-Rad Laboratories, Hercules, CA, USA) as described previously (31) or by a triparental mating method as described previously (46). Kanamycin- or tetracycline-resistant transformants were examined to determine if they were recombinant strains that carried deletion of the gene of interest by PCR. Fluorocytosine (120 µg mL^−1^; Tokyo Chemical Industry, Tokyo, Japan) was used for counterselection of second recombinants: fluorocytosine-resistant strains were examined if they lost the kanamycin or tetracycline resistance. The second recombinant strains were examined to determine if they were the gene deletion mutant or the wild type by checking the length of the gene of interest by PCR as described previously (31).

### Preparation of enzymes

One milliliter of a preculture of *Gluconobacter* strains grown in YPS medium was inoculated into 100 mL YPD medium in a 500 mL Erlenmeyer flask and incubated at 30°C for 16 h with shaking at 200 rpm. The culture was centrifuged at 8,000 × *g* and 4°C for 10 min to sediment the cells; then, the cells were washed with 10 mM K^+^-Pi (pH 6.0) containing 5 mM 2-mercaptoethanol and 300 mM KCl. The cell suspension was centrifuged again to collect the cells and resuspended in 10 mM K^+^-Pi (pH 6.0) containing 5 mM 2-mercaptoethanol and 300 mM KCl in a ratio of 4 mL/g wet cell weight. Potassium chloride was omitted in case of the cells overproduced GLF_0478. Then, phenylmethanesulfonyl fluoride was added to the cell suspension at a final concentration of 0.5 mM, and the suspension was passed through a French cell press (American Instrument Co.) at 1,000 kg cm^−2^, twice. Unbroken cells were removed by centrifugation at 8,000 × *g* at 4°C for 10 min. The supernatant was used as cell-free extract or ultracentrifuged at 100,000 × *g* and 4°C for 1 h to separate the supernatant and precipitate to give the soluble and membrane fractions, respectively. Protein content was determined by the Bradford method (47); bovine serum albumin was used as the standard.

### Enzyme assay

The activity of 2KGR was measured at 25°C spectrophotometrically, by observing the oxidation of NAD(P)H at 340 nm. The reaction mixture consisted of enzyme, 0.1 mM NAD(P)H, 50 mM Na^+^-acetate (pH 6.0), and 10 mM K^+^−2-ketogluconate. Gluconate oxidation activity was measured at 25°C spectrophotometrically, by observing the reduction of NAD(P)^+^ at 340 nm. The reaction mixture consisted of enzyme, 0.1 mM NAD(P)^+^, 50 mM glycine–NaOH (pH 10), and 10 mM Na^+^-gluconate. Absorption coefficients of 6.3 and 6.2 mM^−1^ cm^−1^ were used for NADH and NADPH, respectively. One unit of enzyme activity was defined as the amount that oxidized 1.0 µmol NAD(P)H or gluconate per minute.

### Sequence data retrieval and phylogenetic tree construction

Publicly available Acetobacteraceae genome sequences were downloaded from the NCBI Reference Sequence (RefSeq) FTP website (https://ftp.ncbi.nlm.nih.gov/genomes/refseq/). For phylogenetic analysis, we performed a BLASTP search against all protein-coding sequences from the 434 Acetobacteraceae genomes using the amino acid sequences of GLF_0478 and GLF_1777, homologs of 2KGR GOX0417 of *G. oxydans* 621H (20, 21), as the query (48). The homologous set was selected with a BLASTP filtering expectation value (*e*-value) ≤ 10^−10^ and sequence overlap ≥ 70%. All homologous hits from the two queries were collected, and the merged unique sequence data set was used for phylogenetic tree construction (Table S1). All the collected sequences were aligned using MUSCLE v.3.8.31 at the amino acid sequence level and used for phylogenetic construction (49, 50). The MEGAX 10.2.6 package was used to generate the phylogenetic tree using the neighbor-joining approach with 1,000 bootstrap replicates (51, 52).

## Data Availability

*Gluconobacter* sp. strain CHM43 has been deposited in the NBRC (http://www.nite.go.jp/en/nbrc/index.html) with accession number NBRC 101659. The draft genome sequence has been deposited at DDBJ/EMBL/GenBank under accession no. BADZ02000001 to BADZ02000044. Acetobacteraceae genome sequences used in this study are available publicly at the NCBI Reference Sequence (RefSeq) FTP website (ftp.ncbi.nlm.nih.gov/genomes/refseq/). The data that support the findings of this study are available from the corresponding author upon reasonable request.

## SUPPLEMENTAL MATERIAL

Supplemental figures aem.02501-24-s0001.pdf Figures S1 to S7.

Table S1 aem.02501-24-s0002.xlsx List of 2KGR homologous proteins retrieved with GLF_0478 and GLF_1777 as the queries.

ASM does not own the copyrights to Supplemental Material that may be linked to, or accessed through, an article. The authors have granted ASM a non-exclusive, world-wide license to publish the Supplemental Material files. Please contact the corresponding author directly for reuse.
