# Supplementary material for: Two NADPH-dependent 2-ketogluconate reductases involved in 2-ketogluconate assimilation in Gluconobacter sp. strain CHM43
Source: Appl Environ Microbiol. 2025 Jan 29;91(2):e02501-24. doi: 10.1128/aem.02501-24 (PMC11837542; doi:10.1128/aem.02501-24)
Supplement: Supplemental figures — Figures S1 to S7. [file aem.02501-24-s0001.pdf]

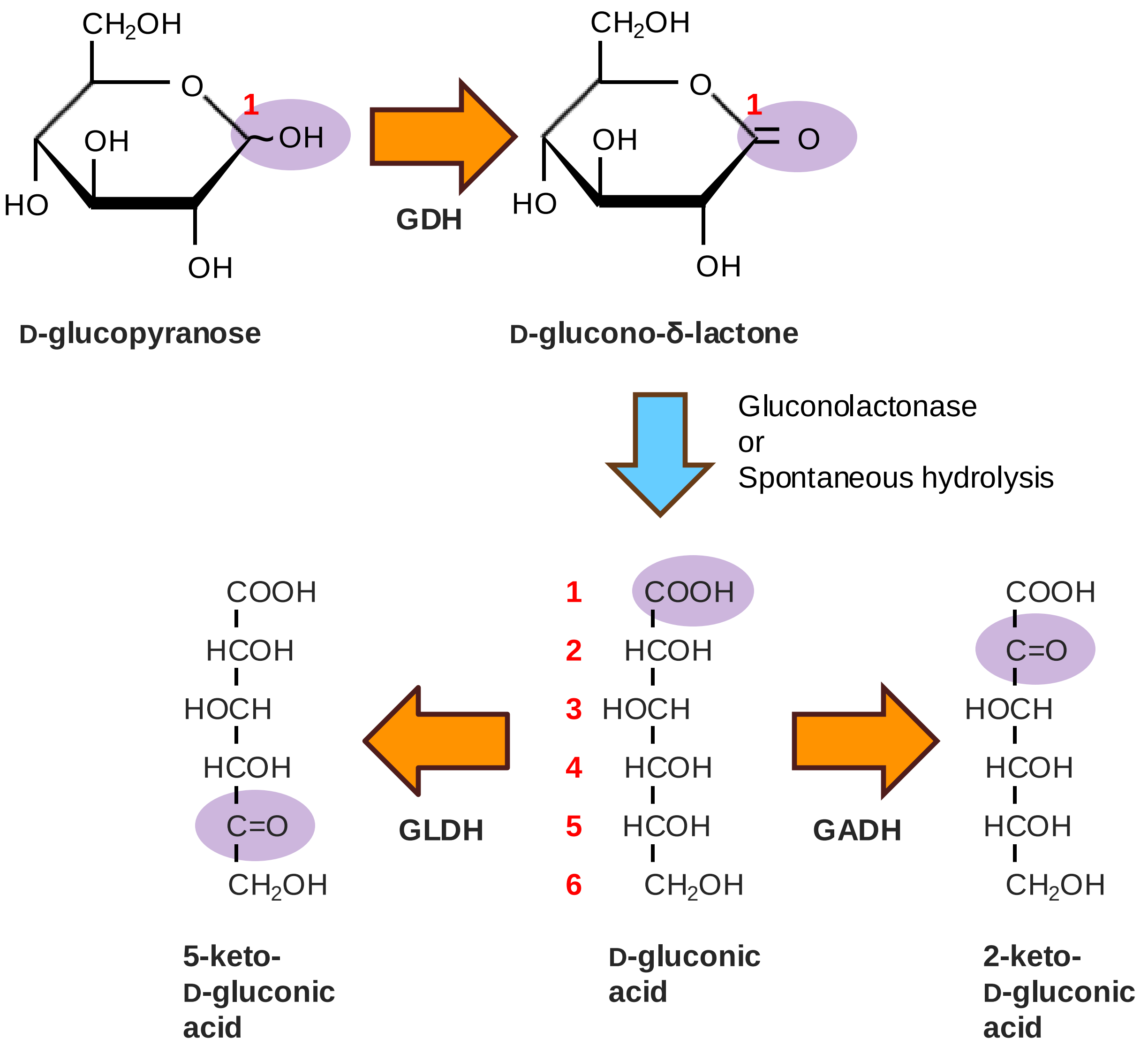

Fig. S1. Chemistry of glucose oxidation by *Gluconobacter* sp. CHM43. Glucose, presumably in the pyranose form, is oxidized to glucono-δ-lactone by glucose dehydrogenase (GDH). Glucono-δ-lactone is hydrolyzed spontaneously or enzymatically by gluconolactonase to gluconic acid. Gluconic acid is oxidized at the 5th position by glycerol dehydrogenase (GLDH) to 5-ketogluconic acid, or at the 2nd position to 2-ketogluconic acid by gluconate 2-dehydrogenase (GADH). Orange and sky-blue arrows indicate oxidation and hydrolysis, respectively.

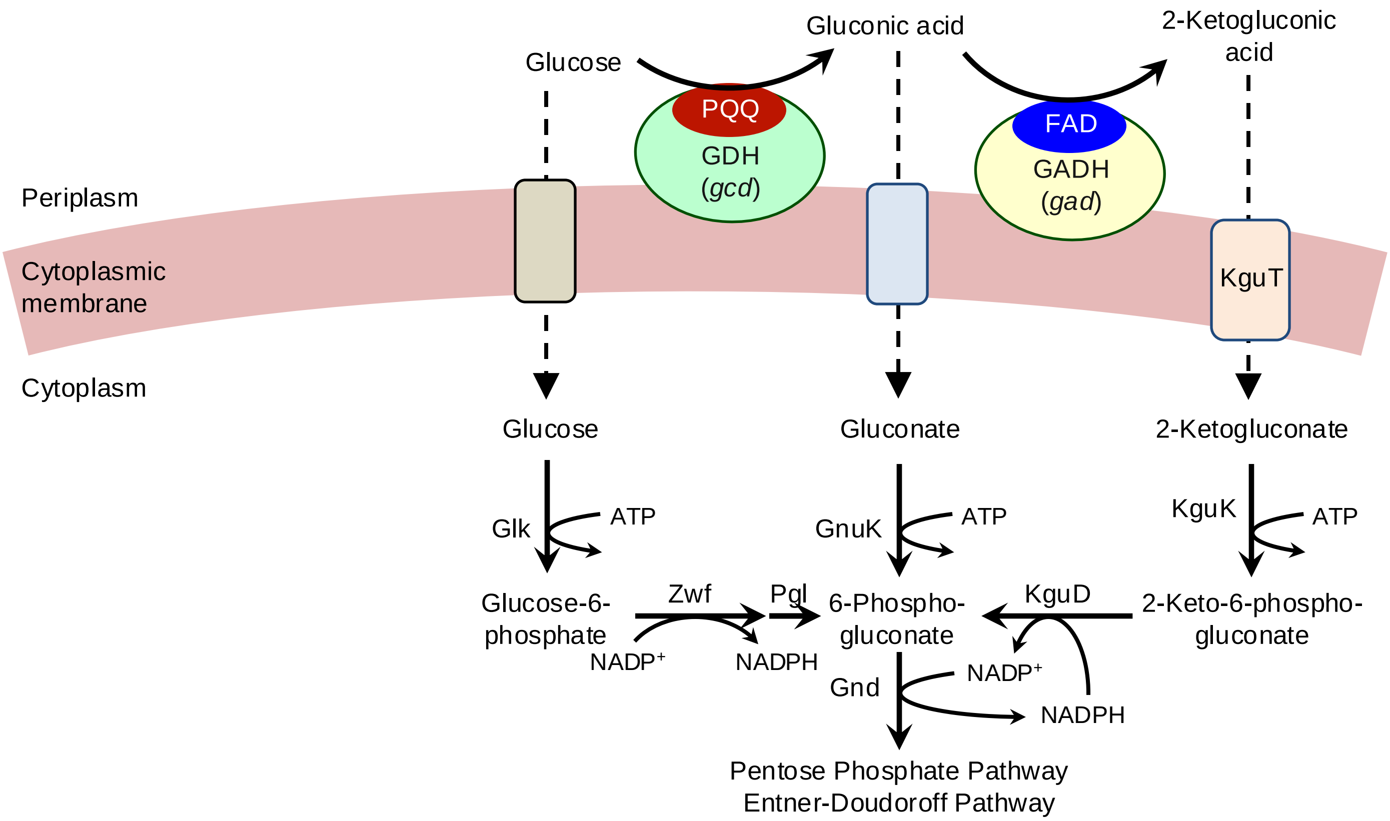

Fig. S2. Metabolic pathway of 2-ketogluconate in *Pseudomonas* spp. GDH, pyrroloquinoline quinone (PQQ)-dependent glucose dehydrogenase encoded by the *gcd* gene; GADH, flavin adenine dinucleotide (FAD)-dependent gluconate dehydrogenase encoded by the *gad* genes; KguT, 2-ketogluconate transporter; KguK, 2-ketogluconate kinase; KguD, 2-keto-6-phosphogluconate dehydrogenase; Glk, glucokinase; GnuK, gluconate kinase; Zwf, glucose-6-phosphate dehydrogenase; Pgl, 6-phosphogluconolactonase; Gnd, 6-phosphogluconate dehydrogenase.

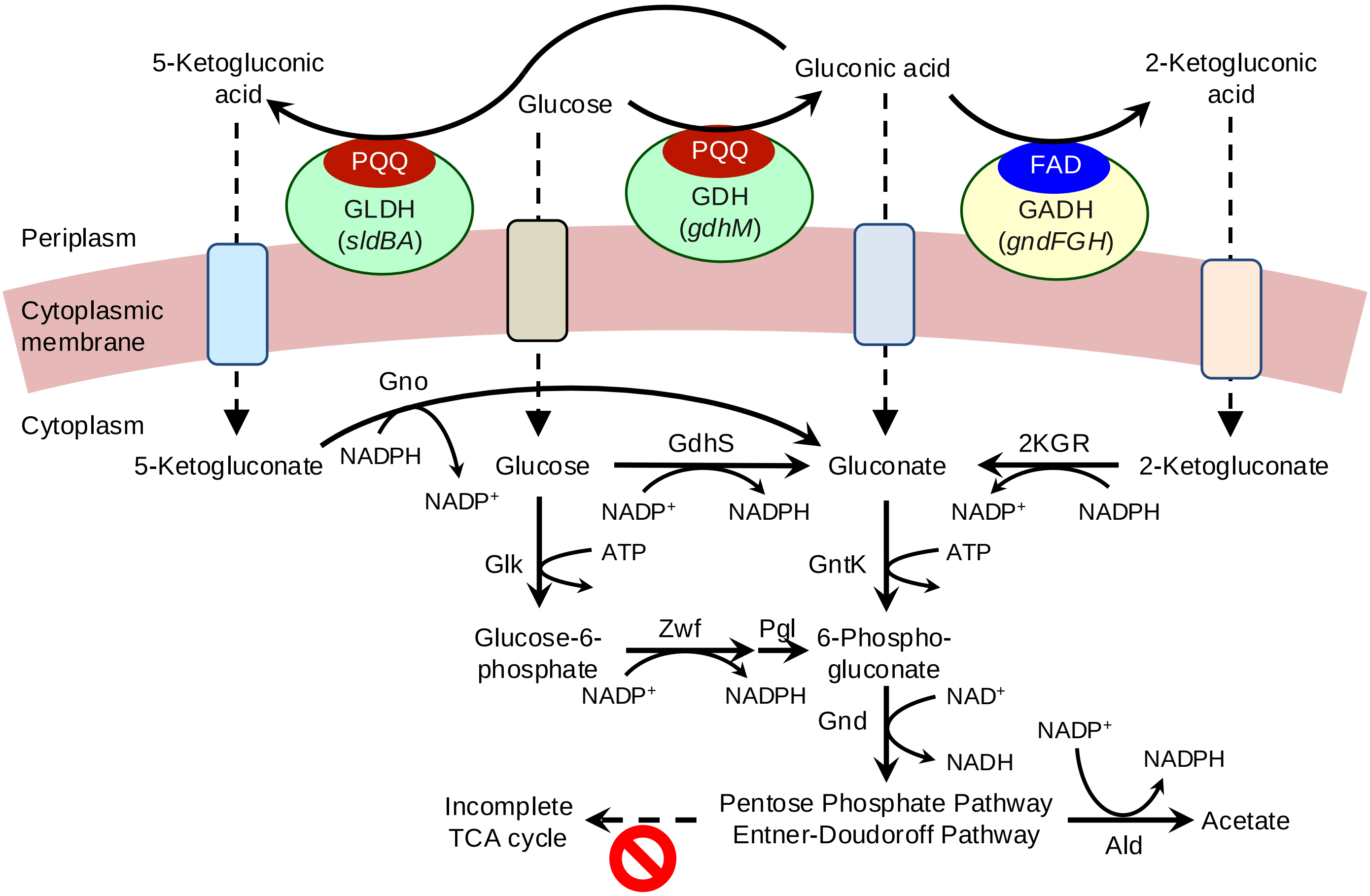

Fig. S3. Proposed metabolic pathways of 2- and 5-ketogluconate in *Gluconobacter* sp. GDH, PQQ-dependent glucose dehydrogenase encoded by the *gdhM* gene; GADH, FAD-dependent gluconate dehydrogenase encoded by the *gndFGH* genes; GLDH, PQQ-dependent glycerol dehydrogenase encoded by the *sldBA* genes, which is responsible for gluconate oxidation to 5-ketogluconic acid; Gno, 5-ketogluconate reductase; GdhS, glucose dehydrogenase; Glk, glucokinase; GntK, gluconate kinase; Zwf, glucose-6-phosphate dehydrogenase; Pgl, 6-phosphogluconolactonase; Gnd, 6-phosphogluconate dehydrogenase; Ald, aldehyde dehydrogenase; 2KGR, 2-ketogluconate reductase.

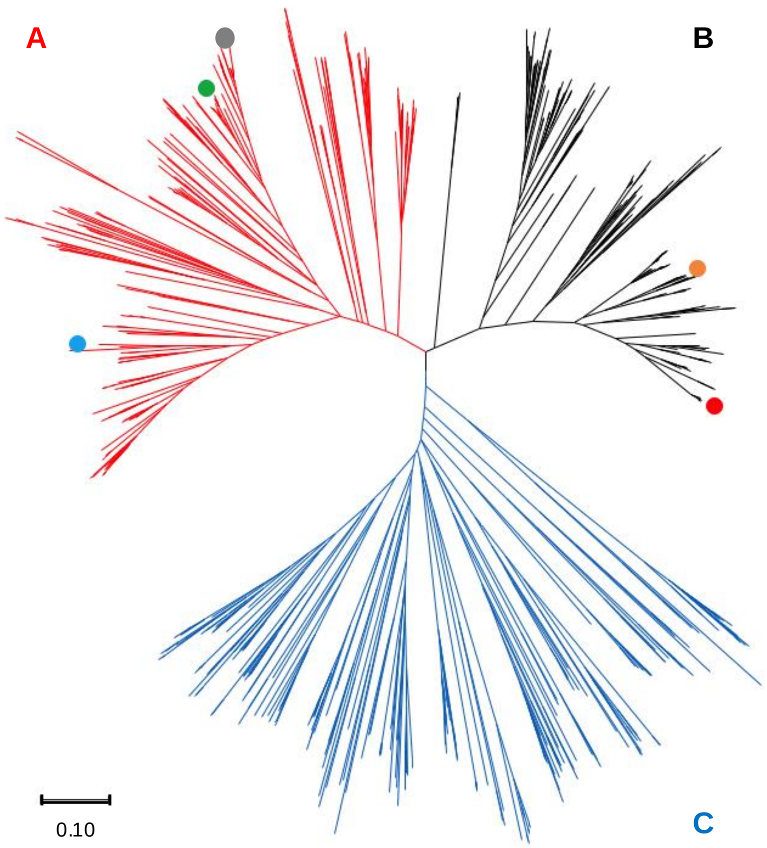

Fig. S4. Unrooted neighbor-joining phylogenetic tree of 2-hydroxyacid dehydrogenases in acetic acid bacteria for which genomes are available. The phylogenetic tree of 978 amino acid sequences was constructed using MEGAX 10.2.6 software. Three clades, "A," "B," and "C" were found in the tree. Circles indicate biochemically characterized 2-ketogluconate reductases (2KGRs). Gray, GOX0417 of *Gluconobacter oxydans* ATCC 621H; orange, GLI01_12690 of *Gluconacetobacter liquefaciens* NBRC 12388; red, NBRC3299_RS04265 of *Acetobacter pasteurianus* NBRC 3299; green, GLF_0478 from *Gluconobacter* sp. CHM43; blue, GLF_1777 from *Gluconobacter* sp. CHM43.

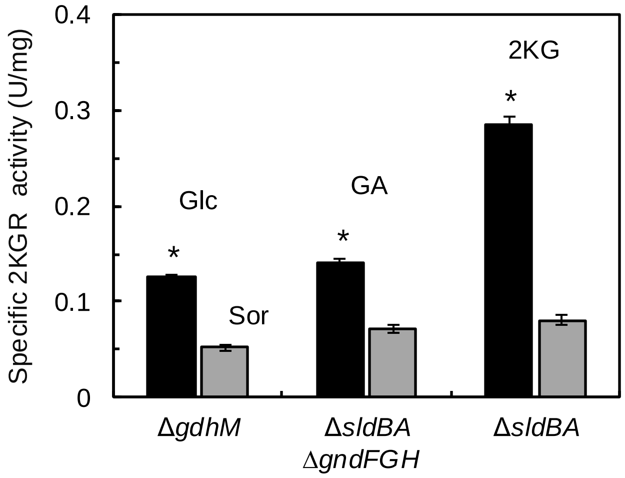

Fig. S5. Induction of 2-ketogluconate reductase. Derivatives of *Gluconobacter* sp. CHM43 were cultivated. The strains UCD1 (Δ*gdhM*) lacks pyrroloquinoline quinone-dependent glucose dehydrogenase and does not oxidize glucose at the periplasmic space. TERA3 (Δ*sldBA* Δ*gndFGH*) lacks glycerol dehydrogenase and flavin adenine dinucleotide-dependent gluconate 2-dehydrogenase and does not oxidize gluconate at the periplasmic space. The UCD1, TERA3, and TORI3 (Δ*sldBA*) were cultivated in YPD medium (containing glucose, "Glc"), GA medium (containing gluconate, "GA"), or 2KG medium (containing 2-ketogluconate, "2KG"), respectively, at 30°C for 16 h with shaking. 2KGR activity in the cell-free extract was measured using NADPH. Mean values and standard deviations (error bars) are shown from triplicate enzyme assays. Asterisk indicates significance to the control conditions, i.e., grown on sorbitol ($P < 0.01$ by Student's t test).

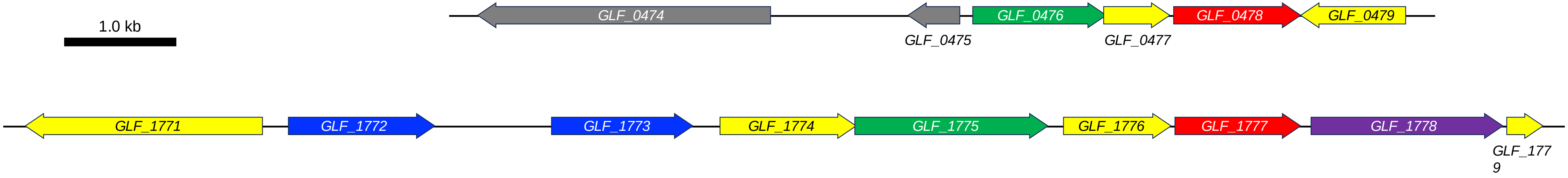

Fig. S6. Organization of the regions adjacent to the genes encoding 2-ketogluconate reductases in the genome of *Gluconobacter* sp. CHM43. GLF_0474 and GLF_0475, hypothetical proteins; GLF_0476, transporter; GLF_0477, protein-tyrosine phosphatase; GLF_0478, 2-hydroxyacid dehydrogenase; GLF_0479, ATP-NAD kinase (PpnK); GLF_1771, sucrose isomerase; GLF_1772 and GLF_1773, transcriptional regulators; GLF_1774, oxidoreductase; GLF_1775, major facilitator superfamily alpha-ketoglutarate/sugar transporter; GLF_1776, 2-oxopent-4-enoate hydratase; GLF_1777, 2-hydroxyacid dehydrogenase; GLF_1778, carbohydrate-selective porin B; GLF_1779, transposase. Red, 2-ketogluconate reductase; gray, hypothetical protein; green, transporter; yellow, enzyme; blue, transcriptional regulator; purple, outer membrane porin.

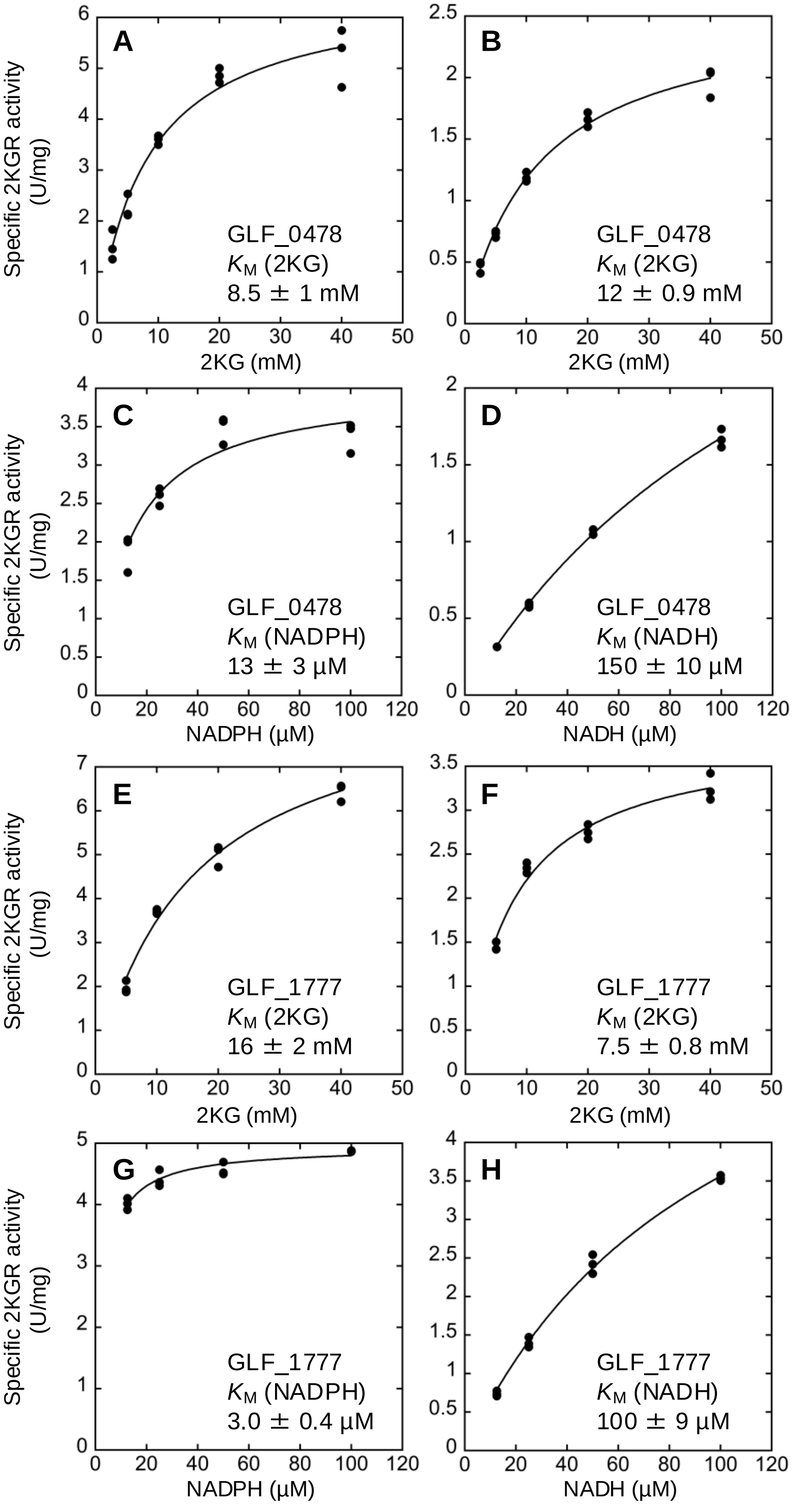
A
GLF_0478
K_M (2KG)
8.5 ± 1 mM
B
GLF_0478
K_M (2KG)
12 ± 0.9 mM
C
GLF_0478
K_M (NADPH)
13 ± 3 µM
D
GLF_0478
K_M (NADH)
150 ± 10 µM
E
GLF_1777
K_M (2KG)
16 ± 2 mM
F
GLF_1777
K_M (2KG)
7.5 ± 0.8 mM
G
GLF_1777
K_M (NADPH)
3.0 ± 0.4 µM
H
GLF_1777
K_M (NADH)
100 ± 9 µM
Specific 2KGR activity (U/mg)
2KG (mM)
NADPH (µM)
NADH (µM)

Fig. S7.

Fig. S7. 2-Ketogluconate reductase activity in recombinant *Gluconobacter* as a function of substrate and cofactor concentrations. Two derivatives of *Gluconobacter* sp. CHM43 were cultivated in YPD medium at 30°C for 16 h with shaking (200 rpm): strain SKR117 (Δ*GLF_1777*) harboring pSKR58 (GLF_0478^{+}) to assay GLF_0478 (A, B, C, and D), and strain SKR104 (Δ*GLF_0478*) harboring pSKR57 (GLF_1777^{+}) to assay GLF_1777 (E, F, G, and H). Soluble crude cell extracts of the recombinant *Gluconobacter* strains were used. A and E, 2KGR activity as a function of 2KG concentration using 0.1 mM NADPH as electron donor; B and F, 2KGR activity as a function of 2KG concentration using 0.1 mM NADH as electron donor; C and G, 2KGR activity as a function of NADPH concentration using 10 mM 2KG; D and H, 2KGR activity as a function of NADH concentration using 10 mM 2KG. K_M values, calculated by using KaleidaGraph (ver. 5.0), are shown on the charts.
